# Visible Light-Induced Degradation of Methylene Blue in the Presence of Photocatalytic ZnS and CdS Nanoparticles

**DOI:** 10.3390/ijms131012242

**Published:** 2012-09-25

**Authors:** Nayereh Soltani, Elias Saion, Mohd Zobir Hussein, Maryam Erfani, Alam Abedini, Ghazaleh Bahmanrokh, Manizheh Navasery, Parisa Vaziri

**Affiliations:** 1 Department of Physics, Faculty of Science, University Putra Malaysia, 43400UPM Serdang, Selangor, Malaysia; E-Mails: maria_395@yahoo.com (M.E.); abedini_alam@yahoo.com (A.A.); ghazalehbahmanrokh@yahoo.com (G.B.); navaseri@gmail.com (M.N.); 2 Department of Chemistry, Faculty of Science, University Putra Malaysia, 43400UPM Serdang, Selangor, Malaysia; E-Mail: mzobir@science.upm.edu.my; 3 Department of Medical Physics and Biomedical Engineering, Shahid Beheshti University of Medical Sciences, Iran; E-Mail: parisa_vaziri@ymail.com

**Keywords:** ZnS, CdS, nanoparticles, photocatalytic, visible light, degradation, methylene blue, pH

## Abstract

ZnS and CdS nanoparticles were prepared by a simple microwave irradiation method under mild conditions. The obtained nanoparticles were characterized by XRD, TEM and EDX. The results indicated that high purity of nanosized ZnS and CdS was successfully obtained with cubic and hexagonal crystalline structures, respectively. The band gap energies of ZnS and CdS nanoparticles were estimated using UV-visible absorption spectra to be about 4.22 and 2.64 eV, respectively. Photocatalytic degradation of methylene blue was carried out using physical mixtures of ZnS and CdS nanoparticles under a 500-W halogen lamp of visible light irradiation. The residual concentration of methylene blue solution was monitored using UV-visible absorption spectrometry. From the study of the variation in composition of ZnS:CdS, a composition of 1:4 (by weight) was found to be very efficient for degradation of methylene blue. In this case the degradation efficiency of the photocatalyst nanoparticles after 6 h irradiation time was about 73% with a reaction rate of 3.61 × 10^−3^ min^−1^. Higher degradation efficiency and reaction rate were achieved by increasing the amount of photocatalyst and initial pH of the solution.

## 1. Introduction

Textile, paper and some other industrial processes discharge large amounts of colored dye wastewater which is toxic and non biodegradable in most cases and when it reaches the natural water runoff, the impending photosynthetic activity of aquatic plants seriously threatens the whole ecosystem. Over the past several decades, various physical, chemical, and biological techniques for decoloration of dye effluents have been developed. Conventional treatments such as coagulation, flocculation, absorption, adsorption, ultrafiltration, reverse osmosis, and membrane technologies merely concentrate or transfer organic compounds from one phase to another. Destructive techniques such as chemical oxidation and advanced oxidation processes (AOPs) may overcome these problems, but still suffer from high costs and incomplete degradation. Among AOPs, one of the most efficient and economical methods is probably photocatalysis under visible-light irradiation, which does not require additional chemicals and the main component in the solar spectra and indoor illuminations is visible light [1–6].

Photocatalysis is a process by which a semiconductor material absorbs light of energy greater than or equal to its band gap, causing excitations of valence band electrons in the conduction band. Such charge separation leads to the formation of electron-hole pairs which can further generate free radicals in the system for redox of the substrate. The resulting free-radicals such as hydroxyl (•OH) are very efficient oxidizers of organic materials and can degrade pollutants [3,7].

For efficient degradation of dyes, a photocatalyst that has a suitable band gap, flat band potential/energy levels and good adsorption properties in the visible region is needed. In both respects CdS with a direct band gap of ~2.4 eV is a promising material whose photocatalytic activity may be improved by activating with ZnS [8,9]. ZnS is a type of II–VI compound semiconductor material with a wide band gap of ~3.7 eV which can absorb light with wavelengths below 380 nm. ZnS nanocrystals are believed to be an effective photocatalyst, resulting from rapid generation of electron-hole pairs and highly negative redox potentials of excited electrons [10–13].

To fully achieve the photocatalysis properties of ZnS and CdS, a well-defined structure is desirable. Generally, the photocatalytic activity of semiconductor materials can be strongly influenced by their structure and particle size, in which particle size is the most important factor [14]. The reactivity of nanosized semiconductors is often altered or enhanced with respect to their bulk counterparts due to size-dependent changes in their redox potentials and high density of the active surface states associated with large surface-to-volume ratio. Moreover, recombination of electron-hole pairs within the semiconductor particle is drastically reduced as particle size decreases [7,15].

The size and structure of nanoparticles depend upon the synthesis methods and conditions (e.g., temperature, pressure, and composition). Amorphous particles can be crystallized if proper treatment is used to meet the energy requirements. In recent years, several synthesis methods have been successfully developed to prepare semiconductor nanostructures including a hydrothermal method [16,17], a solvothermal method [18], a sonochemical method [19,20], gamma-irradiation [21,22], microwave irradiation [23,24] and a micro-emulsion method [25,26]. Among these methods, the microwave assisted route is becoming an increasingly popular method for chemical synthesis. It offers a clean, cheap and convenient method of heating, which often results in higher yields and shorter reaction times and is capable of producing smaller particles with a narrow particle size distribution and high purity [27,28].

Based on previous knowledge of researches, we developed a simple microwave irradiation method for the synthesis of highly crystalline ZnS and CdS nanoparticle semiconductor quantum dots to evaluate their photocatalytic activity. The obtained nanoparticles were characterized by XRD, TEM, EDX, and UV-Vis absorption spectroscopy. The photocatalytic activity of the obtained nanostructures was examined using the degradation of methylene blue (MB) under visible light in air at room temperature by considering the influence of experimental parameters such as the ZnS:CdS weight ratio, the amount of catalyst used and the initial pH of the solution.

## 2. Results and Discussion

### 2.1. Material Characterization

The formation of ZnS and CdS nanoparticles can be observed by the change in color of the solution from colorless to white and yellow respectively and confirmed by powder X-ray diffraction studies.

**Figure 1:**
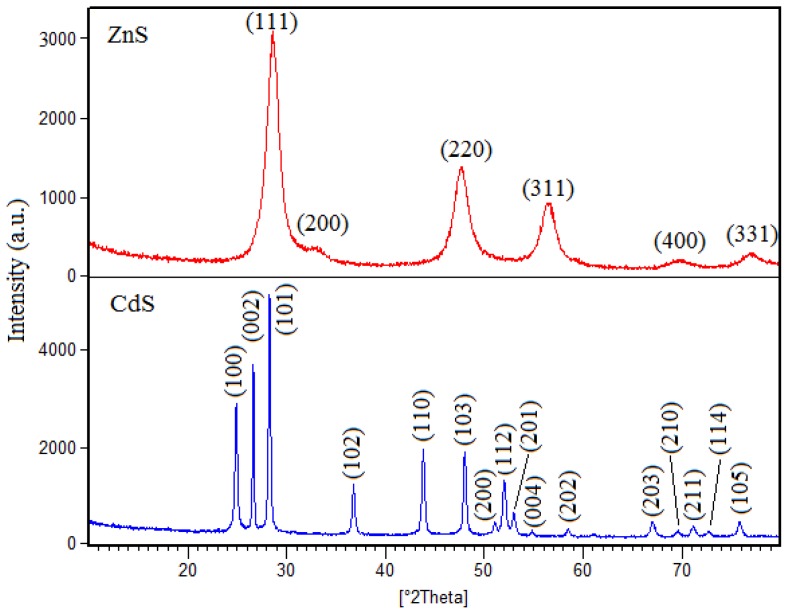
X-ray diffraction (XRD) pattern of the ZnS and CdS nanoparticles.

Figure 1 shows the XRD patterns of ZnS and CdS nanocrystals. In this figure the peaks observed in the XRD patterns of ZnS nanoparticles at 2*θ* values of 28.5°, 33.1°, 47.4°, 56.3°, 69.4° and 76.7°, match perfectly with the (111), (200), (220), (311), (400) and (331) crystalline planes of the face centered cubic structure of ZnS reported in ICDD PDF 65-1691 with lattice parameter of 5.41 A° and cell volume of 158.4 A°^3^. For CdS nanoparticles, the peaks observed in the XRD patterns at 2*θ* values of 24.9°, 26.6°, 28.3°, 36.8°, 43.9°, 48.1°, 51.1°, 52.1°, 53.1°, 54.9°, 58.6°, 67.1°, 69.6°, 71.2°, 72.8° and 75.9° closely match with the (100), (002), (101), (102), (110), (103), (200), (112), (201), (004), (202), (203), (210), (211), (114) and (105) crystalline planes of the hexagonal structure of CdS in the reference pattern of ICDD PDF 80-0006 with the crystal lattice parameters of 4.12, 4.12 and 6.68 A° and cell volume of 98.27 A°^3^. In both patterns no peaks corresponding to impurities were detected, indicating high purity of the products.

**Figure 2:**
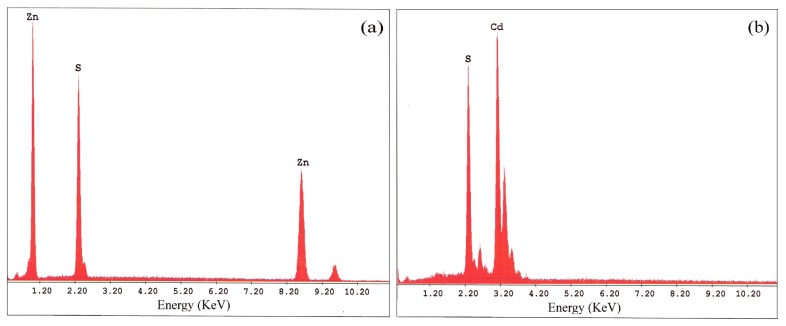
EDX pattern of (**a**) ZnS and (**b**) CdS nanoparticles.

To confirm purity of the products the chemical compositions of ZnS and CdS nanoparticles were analyzed using Energy-dispersive X-ray spectroscopy (EDX). The EDX patterns of nanoparticles are shown in Figure 2 and the related data are listed in Table 1. The results verify the high purity of the nanoparticles.

**Figure 3:**
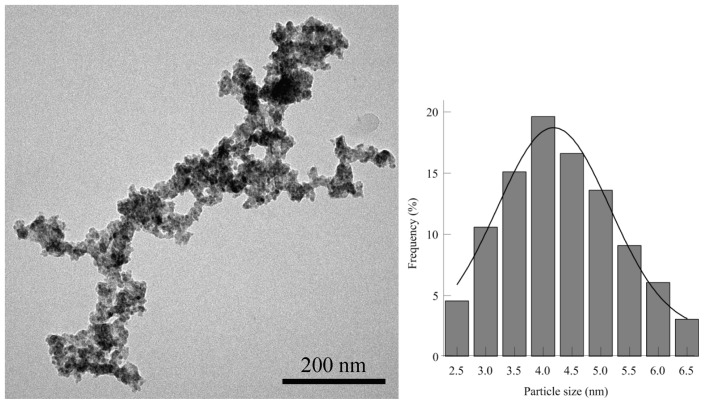
TEM image and particle size distribution histogram of ZnS nanoparticles.

Figure 3 shows the TEM images and corresponding size distribution histogram of ZnS nanoparticles. The figure indicates that ZnS nanoparticles are small spherical particles with a size of less than 7 nm having a relatively narrow homogeneous distribution which appears aggregated. The estimated average particle size is about 4.3 nm.

**Figure 4:**
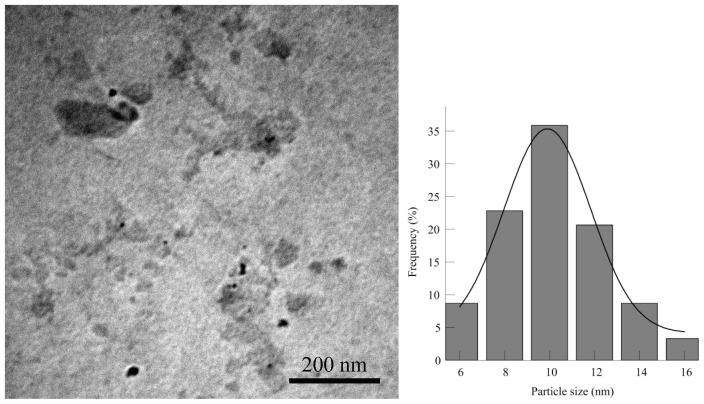
TEM image and particle size distribution histogram of CdS nanoparticles.

The TEM images and corresponding size distribution histogram of CdS nanoparticles are shown in Figure 4. The figure shows that CdS nanoparticles are approximately monodispersed particles of larger sizes, estimated to be between 6 and16 nm with an average particle size of 10.1 nm.

**Figure 5:**
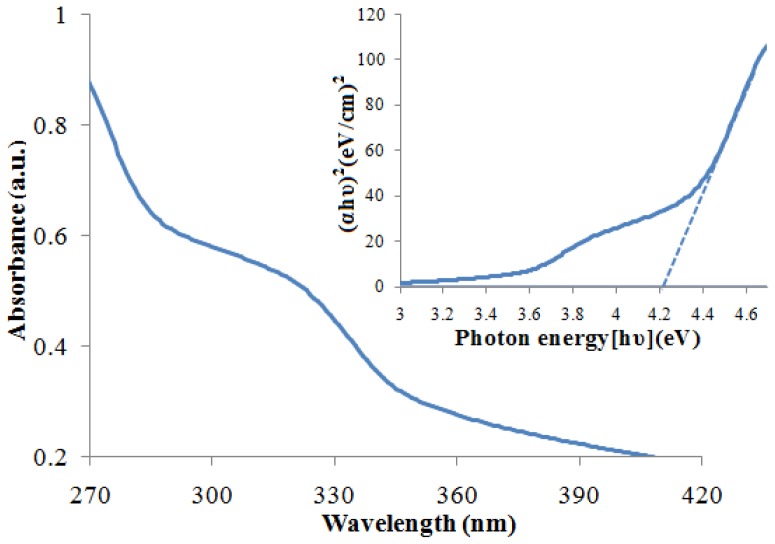
UV–Vis absorption spectra and Tauc plot of the ZnS nanoparticles.

**Figure 6:**
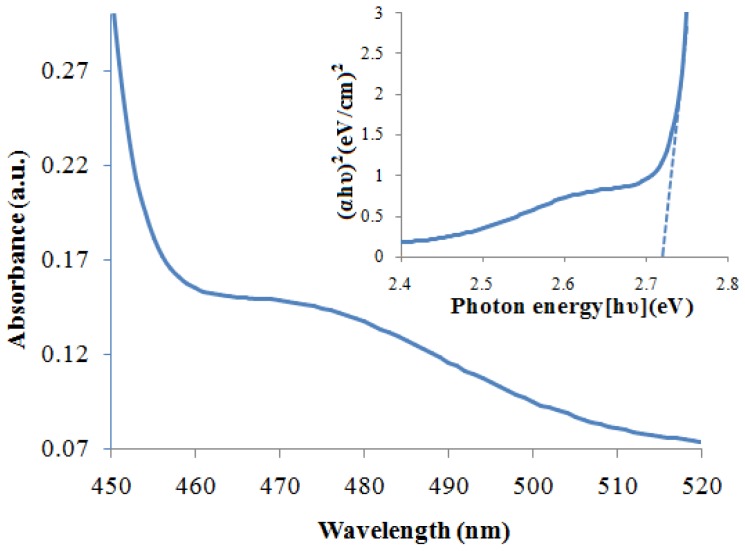
UV–Vis spectra and Tauc plot of the CdS nanoparticles.

The band gap energy (*E*g) of ZnS and CdS nanoparticles can be evaluated from the UV-Vis spectra by Tauc plot of (*hvα*)^2^ versus (*hv*) and extrapolation of the linear portions of the curves to the energy axis according to [29]:

(1) $$\alpha h\nu =B{\left(h\nu -E_{g}\right)}^{1/2}$$

where *α* is the absorption coefficient, *hv* is the photon energy, *E_g_* is the direct band gap energy, and *B* is a constant. The absorption coefficient (*α*) was determined from the relation *A* = *I*/*I*_0_ = *e*^(−^*^αd^*^)^, or it can be calculated using the well-known relation deduced from Beer–Lambert’s relation, *α* = 2.303*A*/*d*, where *d* is the path length of the quartz cuvette and *A* is the absorbance determined from the UV–visible spectrum [30]. UV-Vis absorption spectra and the Tauc plot of ZnS and CdS nanoparticles are shown in Figures 5 and 6. The blue shift of absorption edge compared to their bulk counterparts clearly explains the quantum confinement effect of the nanoparticles. The estimated optical band gaps of ZnS and CdS nanoparticles are about 4.22 eV and 2.64 eV, respectively.

### 2.2. Photodegradation Process

In the presence of air or oxygen, the irradiated semiconductor nanoparticles are capable of destroying many organic contaminants. The activation of ZnS and CdS by light (*hυ*) produces electron-hole pairs which are powerful oxidizing and reducing agents, respectively:

$$\text{ZnS}+h\upsilon \to {\text{h}}^{+}+{\text{e}}^{-}\;\;\;\text{and }\;\;\text{CdS}+h\upsilon \to {\text{h}}^{+}+{\text{e}}^{-}$$

The oxidative and reductive reactions are expressed as:

$${\text{OH}}^{-}+{\text{h}}^{+}\to \cdot \text{OH }\;\;\text{and }\;\;{\text{O}}_{2}+{\text{e}}^{-}\to {{\text{O}}_{2}}^{-}$$

In the degradation of organic compounds (MB), the hydroxyl radical which comes from the oxidation of adsorbed water or adsorbed OH^−^, is the primary oxidant; and the presence of oxygen can prevent the re-combination of hole-electron pairs. For a complete reaction, the final products of the reaction among others are CO_2_ and H_2_O [31,32]:

$$\text{MB}+\;\cdot \text{OH}\to \text{products }({\text{CO}}_{2}+{\text{H}}_{2}\text{O}+{{\text{NH}}_{4}}^{+}\;+\;{{\text{NO}}_{3}}^{-}\;+\;{{\text{SO}}_{4}}^{2-\;}+\;{\text{Cl}}^{-})$$

#### 2.2.1. Influence of Catalyst Composition

**Figure 7:**
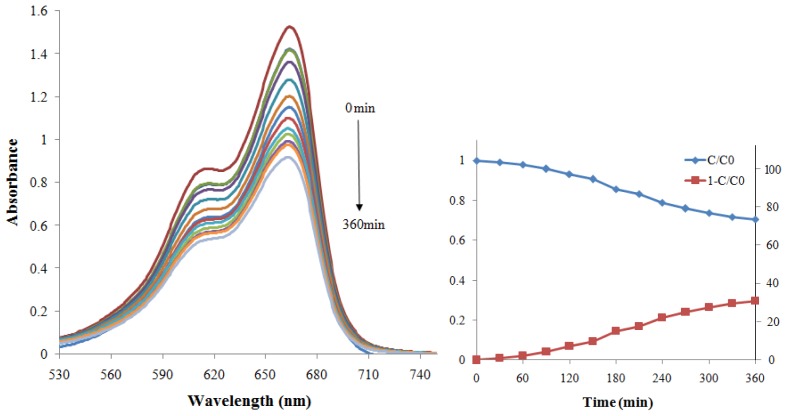
Absorption spectral changes and photodegradation of methylene blue (MB) aqueous solution degraded by ZnS nanoparticles under visible light.

**Figure 8:**
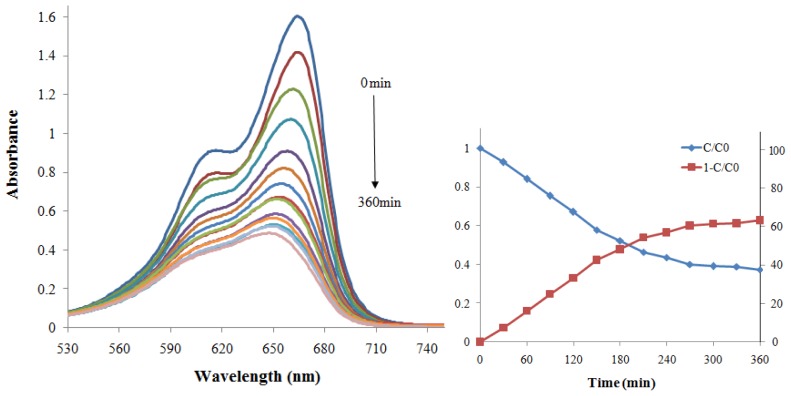
Absorption spectral changes and photodegradation of MB aqueous solution degraded by CdS nanoparticles under visible light.

Figures 7 and 8 show the photodegradation of MB in terms of absorption spectra and as a function of irradiation time in the presence of ZnS and CdS nanoparticles under visible light, respectively. The photodegradation of MB was monitored as the normalized change in its concentration also using degradation efficiency. The intersection of these two curves (*C*/*C*_0_ and 1 − *C*/*C*_0_) shows the half-life of MB, which is the time taken for the concentration of MB to decrease by half. From these figures, it could be seen that the intensity of the adsorption peaks diminished gradually as the exposure time increased, which is more pronounced in the case of using CdS as a photocatalyst. Thereby the normalized concentration change of MB in the presence of CdS nanoparticles is greater than in the presence of ZnS so that for CdS photocatalyst, the concentration of MB has decreased by half after 210 min and the degradation efficiency has reached 63% after 360 min while for ZnS, the efficiency was only nearly 30% after 360 min.

For a photocatalyst to show visible-light activity, a small band gap is also important. Another major criterion for the degradation of organic compound is that the redox potential of the H_2_O/·OH (OH^−^ = ·OH + e^−^; *E*∘ = −2.8 V) couple lies within the band gap of the semiconductor [33].

**Figure 9:**
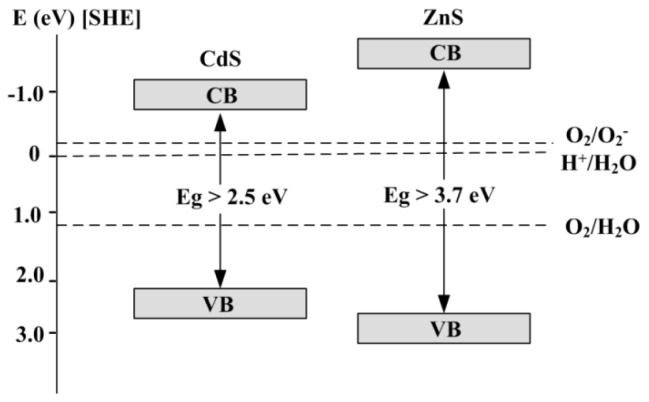
Schematic band structure of ZnS and CdS nanoparticles.

The difference in photocatalytic activities of ZnS and CdS nanoparticles is strongly related to their band gap. As a result of its large band gap, ZnS gives a poor response to visible light, although its conduction band (CB) bottom potential is sufficiently negative (−1.0 eV *vs.* the standard hydrogen electrode (SHE) as shown in Figure 9). In contrast, the small band gap of CdS makes it a good absorber of visible light but the CB bottom potential of CdS is close to that of O_2_/O_2_^−^ (−0.046 eV *vs.* SHE), indicating that it readily undergoes photocorrosion in aqueous media containing oxygen and the over potential is too small to reduce O_2_ with the photoexcited electrons [34], therefore CdS also shows relatively low degradation efficiency (~63% after 6 h). In addition to band gap, the particle size, surface area, crystal structure and degree of crystallinity of ZnS and CdS nanoparticles also have influence on the transportation of photogenerated electrons and holes and subsequently their photocatalyst performance.

**Figure 10:**
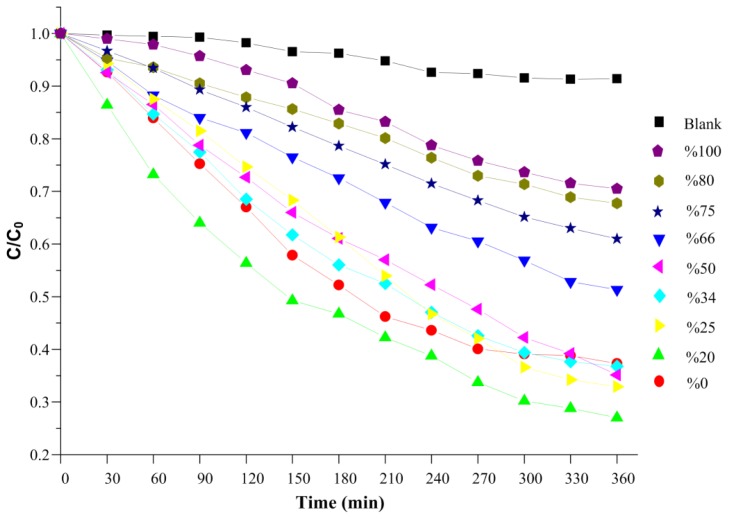
Photodegradation of MB in the presence of ZnS and CdS nanoparticle mixtures with different ZnS weight percentages under visible light.

To increase overall photocatalyst activity of bare ZnS and CdS nanoparticles, a series of experiments was carried out on physical mixtures of them with different weight percentages. For comparison the normalized concentration changes of MB in the absence of photocatalyst but under similar exposure to visible light were measured and compared with those determined in the presence of ZnS and CdS photocatalyst mixture as shown in Figure 10. The related photodegradation data are reported in Table 2.

**Figure 11:**
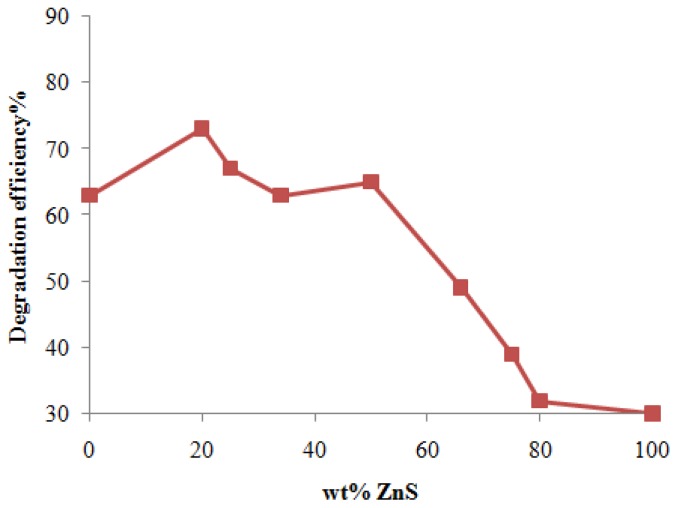
Degradation efficiency of MB in the presence of physical mixtures of ZnS and CdS.

It is apparent from Table 2 that the degradation efficiency of physical mixtures in the degradation of MB has two maxima (Figure 11). It is worth noting that similar results have been reported by other authors for composite CdS-ZnS photocatalysts in hydrogen production [35,36]. The photocatalytic activity increases with decreasing ZnS weight percentage to 50%, then decreases and again increases and reaches its highest amount of 73% for a ZnS percentage of 20% which corresponds to the minimum half-life of 150 min. Accordingly, for the physical mixtures, the photodegradation efficiency should be related to the interaction between the two components and cannot simply be determined by the ZnS weight fraction.

**Figure 12:**
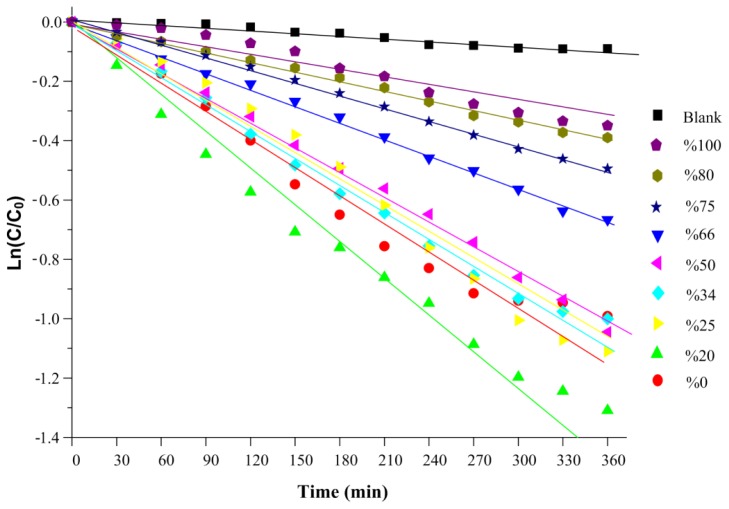
Linear plots of Ln(*C*/*C*_0_) for the photodegradation of MB in the presence of physical mixtures of ZnS and CdS with different ZnS weight percentages under visible light.

Figure 12 shows the linear plots of Ln(*C*/*C*_0_) for the photodegradation of MB with physical mixtures of ZnS and CdS under visible light after 360 min illumination. The slopes of the plots which represent the photocatalyst reaction rate constants were calculated and listed in Table 2. It can be seen from this table that the reaction rate of physical mixtures in the degradation of MB increases with increasing ZnS weight percentage and reaches its maximum at a ZnS percentage of 20%. The maximum reaction rate is 3.61 × 10^−3^ min^−1^.

#### 2.2.2. Influence of Catalyst Amount

**Figure 13:**
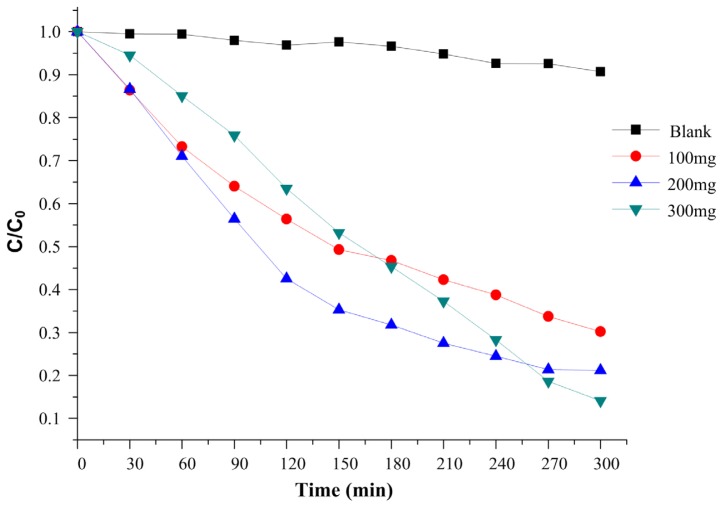
Effect of catalyst amount on the MB decolorization process (ZnS:CdS 20%).

The effect of the catalyst amount on the MB photodegradation (Figure 13) was investigated for ZnS:CdS 20%, since this was the material with the best photocatalytic activity. The rate of MB degradation is dependent on the photocatalyst concentration. With increasing catalyst concentration both the number of dye molecules adsorbed and the number of photons absorbed were increased which promotes the degradation rate. However for very high concentrations of catalyst the suspension’s turbidity increases which decreases the light penetration and consequently the photodegradation is less effective [37].

#### 2.2.3. Influence of the pH

Usually for industrial waste water characteristics, the pH is one of the most important parameters that influences the photo-oxidation process. The effect of the pH on the degradation rate, is due to the modification of the electrical double layer of the solid electrolyte interface, which affects the adsorption-desorption processes and the separation of the photogenerated electron-hole pairs in the surface of the catalyst particles [37].

The surfaces of photocatalysts are positively charged in acidic solutions and negatively charged in alkaline solutions [38]. As a result, the efficiency of the MB photodegradation is expected to increase with pH owing to the electrostatic interactions between the negative photocatalyst’s surface and the MB cations. Moreover, at high pH, the most favorable for sulfur-containing organic oxidation, the photocatalyst corrosion is minimal [39].

**Figure 14:**
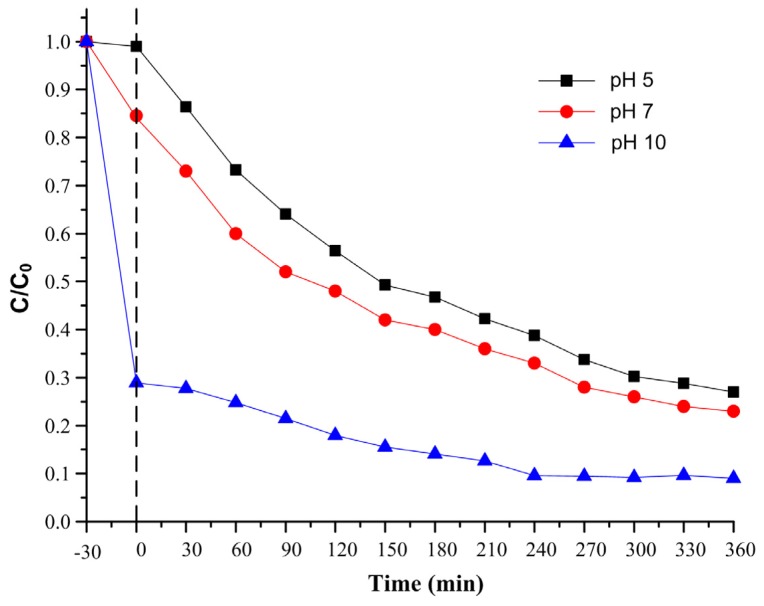
Photocatalytic decolorization of MB solution using different initial pH values (100 mg ZnS:CdS 20%).

It is a well known process in an acidic or alkaline solution that MB degradation is due to the change in hydrogen concentration of the solution towards higher pH. However, in a solution containing MB and quantum dots such as ZnS:CdS used as catalysts, the effect of MB photodegradation does not change with the pH of the solution. To show this we did MB photodegradation experiments in solutions at three different pHs to evaluate the effect of ZnS:CdS catalysts in acidic and alkaline solutions. The ability of the MB to be adsorbed on the photocatalyst’s surface was tested using suspensions of MB and ZnS:CdS (20%) under dark conditions (Figure 14). For pH 5 a slight adsorption onto the photocatalysts surface was observed during light-off (first 30 min) which is due to the cationic behavior of both, ZnS:CdS and MB. Conversely, for pH 10 a very high amount of the dye was adsorbed. For neutral pH an intermediate adsorption situation was observed.

## 3. Experimental Section

### 3.1. Preparation of ZnS and CdS Nanoparticles

The starting materials for the synthesis of ZnS and CdS nanoparticles were zinc acetate (R & M Chemical) and cadmium chloride (Acros Organics) as zinc and cadmium sources respectively, thioacetamide as a sulfur source and ethylene glycol as a solvent. All chemicals were analytical grade products and used without further purification.

In a typical synthesis, 0.005 M of zinc or cadmium source and 0.006 M of sulfur source were added in a glass beaker of 100 mL containing 20 mL of solvent. The solution was stirred at 500 rpm for 30 min. The beaker was placed in a high power microwave oven (1100 W) operated using a pulse regime with 20% power for 25 min irradiation time. The formed precipitates were centrifuged (3500 rpm, 10 min) and the residue was washed several times with distilled water and absolute ethanol. The products were dried in air at 60 °C for 24 h under control environment. The products were characterized by X-ray diffraction (XRD) at a scanning rate of 5°/min in the 2*θ* range 20°–70° using a Philips X-ray diffractometer (7602 EA Almelo, the Netherlands) with Cu Kα radiation (λ = 0.1542 nm). The particle size and size distribution were determined from transmission electron microscopy (TEM) micrographs (HTACHI H-7100 TEM, Chula Vista, CA, USA) operating at 100 keV. The optical properties of ZnS and CdS nanoparticles were characterized using UV–visible absorption spectroscopy (UV-1650PC SHIMADZU, Columbia, MD, USA).

### 3.2. Photocatalytic Reaction

**Figure 15:**
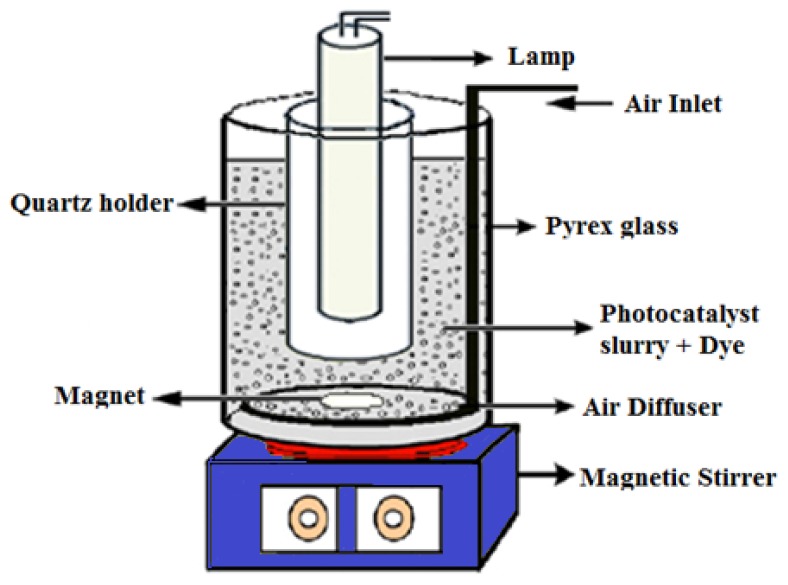
Schematic diagram of the photocatalytic reactor system.

The photocatalytic reactor (Figure 15) is a cylindrical Pyrex-glass cell with 1.0 L capacity. A 500-W halogen lamp as the visible light source (emission range of 400–800 nm) was placed in a quartz lamp holder which was immersed in the photoreactor cell. The cell was filled with 0.6 L of 10 mg/L of MB solution and 100 mg/L of nanoparticles as photocatalyst. The whole reactor was cooled with an electric fan from outside the cell and the temperature was kept at 25 °C. All reactants in the reaction cell were stirred using a magnetic stirrer while fresh air bubbles were introduced into the suspension using a pump. Analogous control experiments were performed without the photocatalyst nanoparticles (blank). The degradation of MB was monitored by taking 4 mL of the suspension at the irradiation time intervals (30 min). Each time the suspension was centrifuged to separate the photocatalyst particles from the MB solution. Subsequently, the degradation rate was calculated according to the change in absorbance of the dye solution.

The absorption spectra of the samples were recorded by measuring the absorbance at 664 nm corresponding to the maximum absorption wavelength of MB with UV–visible absorption spectroscopy. The concentration of MB is proportional to the absorbance of MB according to the Beer–Lambert law, so the degradation efficiency of MB can be calculated by [40,41]:

(2) $$R=\frac{C_{0}-C}{C_{0}}\times 100\%=\frac{A_{0}-A}{A_{0}}\times 100\%$$

where *A*_0_, *A*, and *C*_0_, *C* are the absorbance and concentration of MB when the reaction time is 0 and *t*, respectively. The photocatalytic reaction rate depends on the concentration of the organic pollutants and can be described by the following kinetic model [15]:

(3) $$rate=-\frac{dC}{dt}=\frac{kKC}{1+KC}$$

where *C* is the concentration of MB (mg/L) at any time, *t* is the irradiation time, *k* is the first-order rate constant of the reaction and *K* is the adsorption constant of the pollutant on the photocatalyst. This equation can be simplified to a pseudo-first-order equation [34]:

(4) $$Ln\frac{C}{C_{0}}=-kKt=k_{obs}t$$

in which *k_obs_* is the observed first-order rate constant of the photodegradation reaction. The observed first-order rate constant for the photocatalytic degradation of MB on the nanocrystallines was calculated using plots of *Ln C*/*C*_0_ versus irradiation time.

## 4. Conclusions

This paper presented a simple method for preparation of efficient visible light photocatalytic materials, namely high purity ZnS and CdS nanoparticles, by a microwave irradiation route. The obtained ZnS and CdS nanoparticles had cubic and hexagonal structures respectively with average particle sizes of 4.3 nm and 10.1 nm corresponding to band gap energies of 4.22 eV and 2.64 eV respectively.

Photocatalytic degradation of methylene blue was carried out using physical mixtures of ZnS and CdS nanoparticles at different weight percentages under visible light irradiation. The results show that the photocatalytic activity of the samples depends on the weight percentage of ZnS in the physical mixture. The samples with a ZnS weight percentage of less than 50% show a degradation efficiency equal to or more than pure CdS. The highest photodegradation of methylene blue was observed for a ZnS weight percentage of 20%. In this case the degradation efficiency of the photocatalyst nanoparticles after 360 min illumination was about 73% with a reaction rate of 3.61 × 10^−3^ min^−1^. The photodegradation efficiency was promoted by increasing the concentration of photocatalyst and the initial pH of the solution.

## Figures and Tables

**Table 1:** Energy-dispersive X-ray spectroscopy (EDX) data of ZnS and CdS nanoparticles.

Sample	Element	wt%	At%
ZnS	S	30.63	47.37
	Zn	69.37	52.63
	total	100.00	100.00

CdS	S	21.13	48.44
	Cd	78.87	51.56
	total	100.00	100.00

**Table 2:** Photodegradation data of MB in the presence of ZnS and CdS mixtures.

Sample	ZnS:CdS	wt% ZnS (mg)	wt% CdS (mg)	Half-life (min)	Degradation efficiency%	Rate const. after 360 min (×10^−3^ (min^−1^))
1	(1:0)	100	0	-	30	1.09
2	(4:1)	80	20	-	32	1.11
3	(3:1)	75	25	-	39	1.43
4	(2:1)	66	34	360	49	1.88
5	(1:1)	50	50	270	65	2.89
6	(1:2)	34	66	240	63	2.99
7	(1:3)	25	75	240	67	3.37
8	(1:4)	20	80	150	73	3.61
9	(0:1)	0	100	210	63	2.98
